# S47. ADD-ON SPIRONOLACTONE FOR THE TREATMENT OF SCHIZOPHRENIA (SPIRO TREAT)

**DOI:** 10.1093/schbul/sby018.834

**Published:** 2018-04-01

**Authors:** Astrid Roeh, Stefan Leucht, Peter Falkai, Berthold Langguth, Irina Papazova, Alkomiet Hasan

**Affiliations:** 1 Ludwig Maximilian University; 2 Technical University of Munich; 3 University of Regensburg

## Abstract

**Background:**

Patients with schizophrenia often display three main types of symptoms: positive symptoms (e.g. auditory hallucinations or delusions), negative symptoms (e.g. blunted affect, lack or decline in speech, social withdrawal) and cognitive symptoms (e.g. impairment of working memory and declarative memory). Treatment of positive symptoms with available antipsychotics is well established, while therapy options for cognitive and negative symptoms are lacking. Neurobiological studies identified an enhanced NRG1-ERBB4 signaling as a risk pathway in schizophrenia. Spironolactone was found to function as an inhibitor of the ERBB4 receptor. In Nrg1 type III transgenic mice, spironolactone treatment led to an improvement of schizophrenia-like symptoms (Wehr et al. 2017). This is the first study to investigate an add-on spironolactone treatment in schizophrenia patients for the treatment of cognitive deficits.

**Methods:**

This is a multicenter, randomized, double-blind, parallel (3-groups), longitudinal pilot study including 3 x 27 (81) patients with a clinically stable schizophrenia. Patients are randomized in three groups: one group receives add-on spironolactone 100 mg for three weeks (intervention I), one group receives add-on spironolactone 200 mg for three weeks (intervention II) and one group receives add-on placebo for three weeks (control group). The primary endpoint is the modification of the working memory in dependence to the intervention as investigated with the n-back task (0-, 1- and 2-back). Secondary endpoints include: modification of other cognitive functions (e.g. declarative memory and attention), psychopathology (e.g. PANSS and CDSS), overall level of functioning via GAF and severity of disease via CGI, changes in the number of patients in remission using the Andreasen Criteria, modifications of the inhibitory cortical function in the context of the prepuls paradigm with TMS, evaluating possible dose-differences, spironolactone effects on mRNA levels in peripheral blood lymphocytes (PBMC measures). Statistical analysis of the primary endpoint will be based on the intention-to-treat (ITT) population including all randomised patients using a mixed model ANOVA.

**Results:**

The first patient was recruited in July 2015. Currently 45 patients are included in the trial. The overall tolerance of the medication was satisfactory with 48 reported AE (adverse events). In 3 cases, the causality of the medication was definite, in 4 cases probable and in 5 cases possible. The other cases were probably or definitely not related to the study medication. SAE (serious adverse events) were not reported. The current state of research will be discussed on the conference.

**Discussion:**

This is the first study to describe the effects of an add-on treatment of spironolactone in patients with a clinically stable schizophrenia. Also, it is the first study with a direct target of a biochemically disturbed signaling pathway in schizophrenia patients with a possibly new treatment option in this severe disease.

Funded by Stanley Foundation: (13T-004). EUDRACT: 2014-001968-35, WHO-Clinical-Trials: http://apps.who.int/trialsearch/Trial2.aspx?TrialID=EUCTR2014-001968-35-DE

Reference:

1. Wehr, M.C., Hinrichs, W., Brzózka, M.M., Unterbarnscheidt, T., Herholt, A., Wintgens, J.P., Papiol, S., Soto-Bernardini, M.C., Kravchenko, M., Zhang, M., et al. (2017). Spironolactone is an antagonist of NRG1-ERBB4 signaling and schizophrenia-relevant endophenotypes in mice. EMBO Mol. Med.

